# Perceived risk of infection, ethical challenges and motivational factors among frontline nurses in Covid-19 pandemic: prerequisites and lessons for future pandemic

**DOI:** 10.1186/s12912-023-01653-7

**Published:** 2024-01-02

**Authors:** Parul Chaudhary, Payal, Pooja Nain, Pooja, Pooja Rana, Pooja Verma, Pooja Yadav, Poonam, Prerna, Geetika Kashyap, Rajesh Kumar

**Affiliations:** College of Nursing, All India Institute of Medical Sciences (AIIMS), Rishikesh, Uttarakhand 249203 India

**Keywords:** Nurse, Ethical, Motivation, Infection, Challenges, Covid-19

## Abstract

**Background:**

Infection risk was significant for front-line nurses during the Covid-19 outbreak. The pandemic presented several ethical difficulties and sapped nurses’ drive to labor harder for longer periods. This study evaluates registered nurses’ perceptions of Covid-19 infection risk, ethical dilemmas, and motivating factors.

**Materials and methods:**

During March and April 2022, 400 registered nurses from a newly established tertiary care hospital participated in this cross-sectional exploratory survey. The risk assessment scale, motivation to work scale, and ethical dilemma scale were used to assess the perceived risk of infection, motivational factors and ethical challenges experienced by the nurses. Appropriate descriptive and inferential statistics were applied to compute the results.

**Results:**

76.4% of nurses feared working as a nurse put them at higher risk of infection. Besides the fear of contracting infection, nurses believed they were the source of infection to family members (70.8%) and people around (67.5%). 63.3% of nurses agree that they do not have the right to refuse treatment and every patient has the right to receive optimal care, regardless of age, gender, and medical conditions. Professional obligation to treat patients (72.3%) and sound professional knowledge and experience (83.5%) are important motivating factors to work during the pandemic. Multilinear regression analysis revealed that professional education (95% CI, 3.845 − 0.694, *p* = 0.005), Covid-19 positive status (95% CI,0.455-2.756, *p* = 0.006), and post-Covid-19 hospitalization (95% CI, 1.395–6.978, *p* = 0.003) and duration of hospitalization (95% CI, 0.754-0.058, *p* = 0.022) are independent predictors of higher perceived risk of infection among nurses.

**Conclusions:**

During the pandemic, nurses were afraid to work and faced personal and family risks of contracting the virus. Despite these challenges, they still feel a strong sense of commitment and dedication to providing the best possible care. Nurse administrators need to create a supportive environment that follows ethical principles and meets the needs of nurses to boost their motivation and encourage them to continue working for longer periods.

## Introduction

The Covid-19 virus, also known as the novel coronavirus, is a type of pulmonary inflammation reported outbreak in Wuhan province of China in December 2019. [1] The pandemic has grabbed the whole globe in a firm grip, and every individual faces unprecedented challenges in delivering optimum healthcare [2].

The coronavirus pandemic brought massive changes in every sphere of human life with its circular impact on living, health, economy, and politics. It changed the face of the health industry to fight the mimicker virus [3]. Since the pandemic breakout, healthcare providers have faced enormous challenges, mainly due to scarcity of resources, pandemic preparedness, maintaining social distancing, and many more [4].

The nursing profession during the pandemic became more stressful and demanding, especially after the overwhelming patient load and work intensity, while accommodating swift changes in protocol and management strategies [5]. The combination of high job demand and resource constraints, including the crunch of personal protective gear and disinfection supplies, further intensifies the stress and deteriorates health and well-being significantly to meet the life-threatening condition [6]. The shortage of nurses is further fuelled by quarantined nurses in the pandemic [4, 7]. In addition, meeting the communication needs of their patient’s family members further fuelled the stress and anxiety among healthcare workers [8]. It is apparent that contributing risk factors will likely escalate amidst a pandemic due to the uncertainties surrounding sources, infection rates, severity, and management strategies.

Nurses’ ethical values are developed by their personal background, professional training, life experiences, and regulations of the nursing statutory body of the country/state [9]. The Indian Nursing Council’s code of ethics may not fully address adhering to ethical values while handling pandemics or emergencies. This could lead to higher ethical challenges for nurses, which in turn could lead to job dissatisfaction and a greater likelihood of wanting to quit. Hence, healthcare professionals must prioritize their well-being to provide their patients with the best possible care in the dimensions of ethical values [10].

Research has shown that nurses face ethical dilemmas when dealing with infection risks and lack of protective gear [9]. Nurses risking their health to care for critically ill patients during the pandemic report ethical pressure due to lack of protective gear and high risk of infection [2, 11].

A significant risk of infection has a massive impact on motivation among nurses, and it needs to examine to develop specific psychological interventions to keep the morale and work spirit high for an extended period. This study is crucial in comprehending nurses’ challenges during these difficult times and can help inform future policies and interventions to support them With this in mind, the authors conducted a study investigating how the pandemic has affected nurses’ working profile, motivation, and ethical challenges.

## Materials and methods

### Design, sample, and settings

The cross-sectional exploratory survey was conducted in March-April 2022 in a tertiary care teaching hospital, Northen Himalaya, India. Researchers used purposive sampling techniques to recruit the nurses in this survey. A consent form along with questionnaires was shared with the nurses to participate in the survey. Nurses working in the hospital since the Covid-19 breakout and available during data collection were included in the study. However, nurses diagnosed with any physical or mental illness and under treatment were excluded from the study.

### Instrumentations

The survey utilized three structured questionnaires: the perceived risk of infection scale, ethical dilemmas scale, and motivation scale. Additionally, a pretested proforma was used to collect personal and professional data of the participants.

#### Socio-demographic data

This structured proforma consists of information on age, gender, religion, professional education, professional experience in nursing, and department. In addition, the survey acquired information on hospitalization history due to Covid-19, quarantine status, number of quarantine days, status on Covid-19 testing, training and education on Covid-19, and involvement in ventilated patients. The socio-demographic sheet got validation from microbiology, psychiatric nursing, and public health experts.

#### Risk assessment scale

A self-structured scale comprising 14 items was utilized to assess risk perception among nurses. This scale measured three main areas of concern: the perceived risk of infection at work, the perceived risk of transmitting the infection to others, and the perceived workplace response to Covid-19. Each item was rated on a 5-point Likert scale ranging from strongly agree (5) to strongly disagree (1); a higher score indicates a higher risk of infection. The scale was validated by psychology, medicine, and nursing experts and was reliable, with a Cronbach’s alpha of 0.82 for this study.

#### Ethical dilemma assessment scale

A 10-item self-structured scale explores ethical challenges nurses face during the Covid-19 pandemic. Each item was measured on a 3-point scale: totally not true (1) to very true (3); a higher score indicates more ethical dilemmas. The scale consists of items on different situations that posed ethical challenges to nurses in the Covid-19 pandemic, including the right to refuse to treat patients, priority to treat coronavirus patients, political pressure to intervene in the treatment of patients, and avoid treating old and geriatric patients over preference to younger patients with coronavirus. The scale sought validation by medicine, epidemiology, and nursing experts. The scale reliability was calculated using Cronbach’s alpha (α = 0.88) and found reliable.

#### Motivation to work scale

An eight-item self-structured motivation scale is prepared, including possible factors influencing nurses’ motivation to work. The items include the role of health status in working, resources availability, leave and wages, feeling obliged to work during the pandemic, attitude of society to nurses during the pandemic, and recognition in the system. Each item was measured on a 5-point scale: not at all true (1) to very true (5); a higher score indicates higher motivation. The scale sought validation by the experts, and the scale’s reliability was measured using Cronbach’s alpha (α = 0.87) for the study. The scale was found valid and reliable to use in this study.

#### Ethical consideration

A short proposal was submitted to the Institutional Ethics Committee (IEC) of All India Institute of Medical Sciences (AIIMS) Rishikesh for approval (XXXX/IEC/22/147). All methods were performed in accordance with the relevant ethical guidelines and regulations. Individual participant was explained about the duration and purpose of the research and their involvement. Before participating in the research, each participant asked to provide an informed consent, with privacy and confidentiality ensured throughout every study stage. A self-structured questionnaires were handed over to the participants to provide relevant information on the study variables. It took around 15–20 minute for each participant to respond to the survey questionnaires. After carefully reviewing the filled questionnaires, 400 were found suitable for inclusion in the final analysis.

### Statistical analysis

The survey questionnaire was shared with 600 registered nurses, of which 420 (70.00%) responded. A datasheet is prepared in Microsoft Excel and analyzed using IBM SPSS Statistics for Windows, Version 23.0. Armonk, NY: IBM Corp. [12]. Descriptive statistics, frequencies, and percentages are used to describe the characteristics of the participants. The data distribution was checked using the Shapiro–Wilk test. Mean and standard deviation is used to compare the findings between different study groups. Various tests were applied to find the association of study variables with participants’ characteristics, including the Kruskal-Wallis Test, Mann-Whitney, independent sample t-test, Pearson coefficient correlation, and Spearman correlations.

## Results

### Participant characteristics

Table 1 provides information on the personal and professional characteristics of the participants. 600 questionnaires were distributed offline, of which 420 were returned to the researchers, and 400 were found suitable to include in the final analysis. Most participants were male (58%) and Hindu (90%).

**Table 1:** Participants characteristics (n = 400)

Age (years)	28.49 (average), 23–40 (range), 2.90 (SD)
Gender	58% male; 42% female
Religion	90% Hindu; 5.6% Muslim, 4.4% Christian
Marital status	52.8% married; 47.2% unmarried
Highest degree in nursing	14.5% diploma; 84% bachelor; 1.5 master
Type of educational institute	49.7% government; 47% private; 3.3% trust or missionary
Professional experience in nursing (years)	4.33 (average), 1–14 (range); 2.29 (SD)
Experience in the current institute (years)	2.84 (average); 1–8 (range); 1.27 (SD)
Department type	18.7% emergency and trauma; 54.5% Covid-19 ICUs; 26.8% general ward
Training and education on Covid-19	88.8% yes; 11.2% no
Involved in care of ventilated patients	93.8% yes; 6.2% no
Tested Covid-19 positive	55.2% yes; 44.2% no
Covid-19 related hospital admission	10.5% yes; 89.5% no
Quarantine status	62.0% yes; 38% no
Quarantine duration (days)	7.26 (average); 6.68 (SD)
Clear management policy in the working area	93.8% yes; 6.2% no
Regular updates on changes in policy/protocol	95.8% yes; 4.2% no

The participants had an average age of 28.49 (SD = 2.9) years. Most of them were married (52.8%), had completed graduation in nursing, and had a total professional experience of 4.33 (SD = 2.27) years and 2.83 (SD = 1.27) years of experience at their current institution. More than half of the frontline nurses (54.5%) worked in the Covid-19 -intensive care unit and attended some training (88.8%) about treatment and infection prevention while dealing with Covid-19 patients.

Also, 55.2% of the participants reported contracting Covid-19 infection and were hospitalized (10.5%) for advanced treatment. More than half (62%) of participants went into quarantine after contracting Covid-19 infection with an average of 7.26 (SD = 6.68) days. The majority of participants (93.8%) were involved in the care of Covid-19 patients and reported that their area had clear policies and guidelines for patient management and got periodic updates (95.8%) on change or revision of treatment and management for Covid-19 patients (Table 1).

### Perceived risk of infection

About the perceived risk of being infected with Covid-19, 76.4% of the participants agreed that working as a nurse put them at a high risk of contracting infection. Also, 59.5% of the participants felt fearful of being infected with the virus. 70.8% of participants agreed that their family believed that they were at high risk of Covid-19 exposure. On the other hand, 65.8% of the participants agreed that nursing jobs put them at high risk of Covid-19 exposure. Further, 57.7% of participants agreed that people around them were worried about their health and 67.5% agreed that people around them were worried that they might contract Covid-19 from nurses.

Regarding the perceived response of the workplace to Covid-19, there was no much difference in the opinions of nurses; 24.8% of the participants strongly believed that they should not take care of Covid-19 patients in contrast to 35.7% who were interested in working at a current health facility. For 37.8% of the participants, it was usual for their colleagues to quit their jobs because they feared Covid-19. Also, 51.5% of the participants expected that their hospital would provide necessary medical services in case of infection (Table 2).

**Table 2:** Perceived risk of infection among frontline nurses (n = 400)

Items	Agree* f(%)	Undecided f(%)	Disagree* f(%)
**Perceived risk of transmitting Covid-19**			
Concerned about transmitting the virus to family members.	310 (77.5)	46 (11.5)	44 (11)
Concerned about transmitting the virus to neighbour.	310 (77.5)	59 (14.8)	31 (7.7)
Concerned about transmitting the virus to community.	286 (71.5)	51 (12.7)	63 (15.8)
Concerned about transmitting the virus to my close colleagues.	295 (73.7)	69 (17.3)	36 (9.0)
**Perceived risk of being infected with Covid-19**			
Nursing jobs put me at high risk of infection.	306 (76.4)	60 (15.0)	34 (8.6)
Afraid of being infected with the virus.	238 (59.5)	109 (27.2)	53 (13.3)
Family believes that I am at high risk of Covid-19 exposure.	283 (70.8)	61 (15.2)	56 (14.0)
My job puts people near me at high risk of Covid-19 exposure.	163 (65.8)	93 (23.2)	44 (11.0)
People around me are worried about my health	231 (57.7)	98 (24.5)	71 (17.8)
People around me are worried about contracting infection from me.	270 (67.5)	79 (19.8)	51 (12.7)
**Perceived response of the workplace to Covid-19**			
I should not take care of Covid-19 patients.	99 (24.8)	48 (12.0)	253 (63.2)
I disagree to continue working at a current health facility since it may infect me.	143 (35.7)	73 (18.3)	184 (46.0)
I accept it normal if my colleague quit job because of afraid of Covid-19.	151 (37.8)	97 (24.2)	152 (38.0)
I believe my hospital would not provide me necessary medical services in case of infection.	109 (27.3)	85 (21.2)	206 (51.5)

*Agree (Agree + strongly agree); Disagree (Disagree + strongly disagree)

Further, it has been reported that male’s nurse had a high apprehension of contracting infection (p = 0.028) compared to their counterparts. The risk of infection was significantly high among nurses who completed a diploma (*p* = 0.005) as a professional education, which need immediate attention to organize in-service education, teaching, and training program for nurses. In India, the diploma and graduate nursing program curriculum has a gap in teaching content, training, and other academic activities, which might be a probable reason to perceive a higher risk of infection in diploma nurses. Finally, nurses who tested Covid-19 positive (p = 0.001) and were hospitalized for advanced care (p = 0.005) for a longer duration (p < 0.05) reported a higher risk of infection than their counterparts (Table 3).

**Table 3:** Participants’ characteristics and risk of infection, ethical dilemma, and motivation status

Variables	Risk of infection (Mean ± SD)	Ethical dilemma (Mean ± SD)	Motivation level (Mean ± SD)
**Gender**			
Male	49.92 ± 5.53*	16.27 ± 2.86	24.14 ± 4.73
Female	48.63 ± 6.03	16.47 ± 2.75	23.82 ± 4.14
**Religion** ^#^			
Hindu	49.45 ± 5.79	16.27 ± 2.77	24.03 ± 4.52
Muslim	50.62 ± 5.85	16.75 ± 2.81	24.50 ± 5.72
Christian	46.82 ± 5.02	17.65 ± 3.52	22.54 ± 3.26
**Marital status**			
Married	49.31 ± 6.08	16.01 ± 2.72*	23.91 ± 4.52
Unmarried	49.46 ± 5.12	16.74 ± 2.87	24.12 ± 4.52
**Professional education**			
Diploma in nursing	51.18 ± 5.13*	15.66 ± 2.96	23.88 ± 3.95
Graduate degree	49.07 ± 5.83	16.47 ± 2.77	42.03 ± 4.62
**Working area**			
Emergency and ICUs	49.15 ± 5.89	16.40 ± 2.68	24.15 ± 4.40
General ward	50.00 ± 5.41	16.22 ± 3.15	23.62 ± 4.80
**Training and education on Covid-19**			
No	50.02 ± 3.99	15.86 ± 2.96	23.27 ± 5.05
Yes	49.30 ± 6.00	16.41 ± 2.79	24.09 ± 4.44
**Involve in care of ventilated patients**			
No	51.16 ± 4.08	17.84 ± 1.68*	25.28 ± 3.46
Yes	49.26 ± 5.85	16.26 ± 2.85	23.92 ± 4.57
**Test Covid-19 positive**			
No	48.29 ± 5.64*	16.46 ± 2.87	24.60 ± 4.28
Yes	50.25 ± 5.74	16.25 ± 2.76	23.50 ± 4.66
**Hospitalization after Covid-19**			
No	49.06 ± 5.59*	16.32 ± 2.79	23.83 ± 4.4
Yes	51.93 ± 6.60	16.66 ± 2.97	25.47 ± 5.07
**Quarantine status**			
No	49.00 ± 5.87	16.58 ± 2.76	24.86 ± 4.17*
Yes	49.62 ± 5.71	16.22 ± 2.84	23.47 ± 4.66
Age (in years)^	-0.009	-0.021	-0.103*
Hospitalization days^	0.141**	0.035	0.161**
Professional experience (in years)^	0.016	-0.088	-0.127
Quarantine days^	0.078	-0.037	-0.190

^-Spearman Rho; #-Krushkal Wallis test; ICU-Intensive care unit; **-p-value < 0.01; *-p-value < 0.05

In Multilinear regression analysis, the findings revealed that professional education (95% CI, 3.845 − 0.694, *p* = 0.005), Covid-19 positive status (95% CI, 0.455-2.756, *p* = 0.006), and hospitalization (95% CI, 1.395–6.978, *p* = 0.003) and duration of hospitalization (95% CI, 0.754-0.058, *p* = 0.022) are independent predictor of higher perceived risk of infection among nurses (Table 4).

**Table 4:** Multiple linear regression analysis for predictors of risk of infection

Model	B	SE	Beta	p-value	95% CI
(Constant)	49.488	0.841		0.000	47.83-51.1415
Gender	1.331	0.566	0.114	0.019	0.217–2.444
Education	-2.269	0.801	− 0.139	0.005*	-3.845-0.694
Covid-19 positive	1.606	0.585	0.140	0.006*	0.455-2.756
Hospitalization (yes)	4.186	1.420	0.227	0.003*	1.395–6.978
Hospitalisation (days)^	− 0.406	0.177	− 0.173	0.022*	− 0.754-0.058

*p < 0.05; SE-standard error; ^-duration of hospitalization

### Ethical dilemma experienced by nurses

**Fig. 1:**
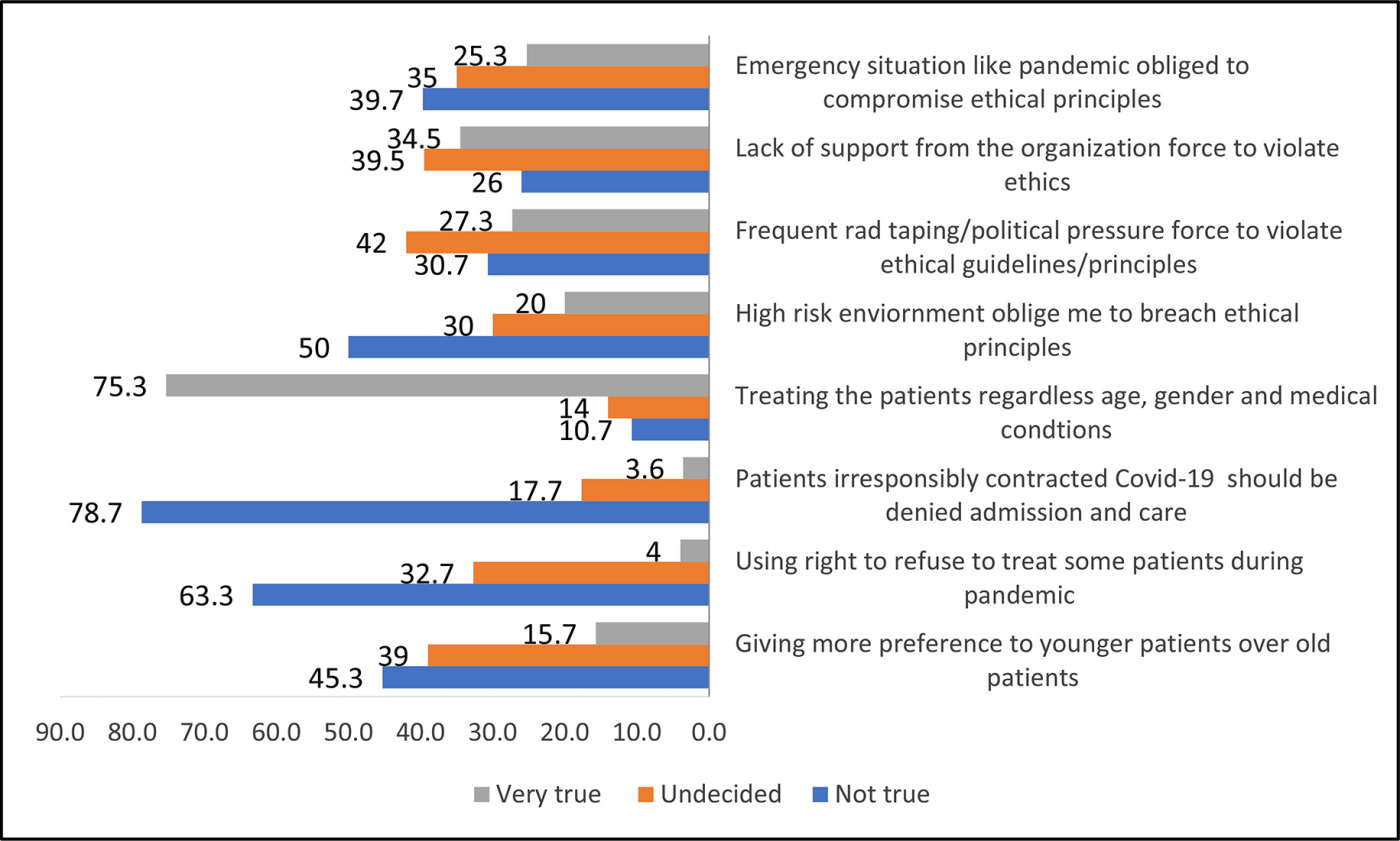
Ethical dilemma among nurses during Covid-19

The response to different ethical dilemmas experienced by nurses during the COVID-19 pandemic revealed that nurses do not believe that they (63.3%) have the right to refuse to treat patients during the Covid-19 outbreak. When asked about medical and treatment resource shortages during the pandemic, around half of the participants (45.3%) disagreed with treating younger patients over older ones. More than three-fourths (75.3%) of participants believed that all patients have equal rights and should be given equal opportunity to be treated during pandemics, regardless of age, gender, and medical background. Similarly, the majority of participants (78.7%) believed that patients who contracted Covid-19 infection after indulging in high risk, i.e., smoking and not abiding Covid-19 appropriate behaviour, should not be denied care. Further, half of the participants (50%) believed that working in a high-risk environment is not a true justification for disregarding ethical principles while caring for patients and compromising the ethical principles (39.7%) during an emergency situation (Fig. 1).

Further, the nurses involved in the care of ventilated patients reported higher odds of ethical dilemmas (*p* = 0.002) considering the difficult decision-making circumstances in the pandemic. These findings indicate more ethical confusion while providing care with scarce resources to overwhelming patient load and being obliged to breach many ethical principles during the pandemic (Table 3).

Further, it was found that a significant proportion of them (27.3%) believed that political pressure or red tape compelled them to breach ethical principles during the pandemic. Additionally, a considerable number of respondents (34.5%) reported that working without adequate monetary compensation or work appreciation also forced them to provide compromised care to Covid-19 patients.

More than half the nurses (61.3%) believed that it is highly untrue that their healthcare organization did not respect their work decisions during the Covid-19. Indeed, management accepted participants’ request for choosing a department to work and shift selection. On the contrary, 23.2% of nurses believed that their demands for choosing work department and shift selection go unheard in the pandemic. Furthermore, 83.5% of the participants believed their knowledge and skills boosted their motivation to work during the pandemic. Also, the most crucial factor included respondents’ professional obligation (72.3%) to care for the patients during the pandemic, the need to work to make a living (70.0%), salary (69%), and encouraging attitude of society towards health professionals (61.6%) in the pandemic.

Disgracefully, 19.7% of nurses reported that society’s negative attitude during the pandemic was discouraging to work. Also, it was unpromising to hear that participants were not offered any extra leave to complete the quarantine period (38.3%) after contracting infection, which was a massive disappointment to work with the same zeal and enthusiasm. (Table 5).

**Table 5:** Perceived motivational factors among frontline nurses (n = 400)

Items	Not True f(%)	Slightly True f(%)	True f(%)
Lack of autonomy in decision making discourages to work.	245 (61.3)	62 (15.5)	93 (23.2)
My knowledge and experience motivate me to work.	17 (4.3)	49 (12.2)	334 (83.5)
My profession obliged me to provide care in pandemic.	57 (14.2)	54 (13.5)	289(72.3)
Matter of survival to work in pandemic.	39 (9.8)	81 (20.2)	280(70.0)
Negative attitude of society demotivates to work.	246 (61.6)	75 (18.7)	79 (19.7)
Working for extra hours with no extra remuneration.	284(71.0)	52 (13.0)	64(16.0)
Low salary/wages stop working in Covid-19.	276(69.0)	75 (18.7)	49 (12.3)
Counting quarantine days in duty hours is demotivating.	182 (45.5)	65 (16.2)	153(38.3)

Further, the pandemic significantly shaken the motivation of nurses with advanced age (r=-0.103, *p* < 0.01). Likewise, higher experienced nurses significantly reported lower motivation (r=-0.127, *p* < 0.05) than the younger brigade. Interestingly, nurses quarantined after contracting infection significantly reported higher motivation *(p* = 0.004), which was again higher in nurses’ hospitalization for longer days (r = 0.161, *p* < 0.05). However, the long quarantine period significantly reduces nurses’ motivation (r=-0.190, p < 0.01). The only factors that influenced the motivation of the nurses were quarantine and hospitalization, which can be postulated that nurses might have considered it a much-needed break from their untiring job during the pandemic to make them motivated to work for a longer time (Table 3).

## Discussion

This study reflects the risk of contracting infection, ethical challenges, and motivation factors of front-line nurses working with Covid-19 patients. For this, researchers use structured pretested proforma to assess the risk of infection, ethical challenges, and motivation to work. We found that more than half of the nurses (55.2%) screened positive for Covid-19 during the pandemic, and more than one-third were apprehended to work with Covid-19 patients. It is understandable that working with Covid-19 patients puts them at a higher risk of contracting infection. It is a known phenomenon that working with Covid-19 patients is highly frightening and remains a matter of safety for nurses [13, 14]. Other studies from India and around the world report similar findings on the fear and anxiety experienced by nurses during the Covid-19 pandemic [15–17]. This highlights the need for urgent attention and action.

Based on this research, several findings align with previous studies on anxiety. For instance, a study in Israel revealed that one-third of nurses were scared to go to work and felt unsafe in their workplace. These results indicate a growing concern for the safety and protection of healthcare workers [9]. According to the research, it was found that nurses expressed fear and concern about caring for patients with Covid-19. Many of them would choose to work with non-Covid-19 patients instead. This highlights the need for additional safety measures and support for healthcare workers during the pandemic [9]. Similarly, another study reported a higher prevalence of anxiety among nurses working with Covid-19 patients [18]. On the contrary, a study conducted in India reported a low prevalence of anxiety [19]. Authors has speculated that the varying results on anxiety among nurses could be attributed to the fluctuating Covid-19 cases in India and the loosening of restrictions during different waves in the latter stages. It is possible that initial feelings of panic, fear, and anxiety may not align with accurate reporting of information regarding the virus. However, as more information becomes available from the Ministry of Health, real-time data updates, media awareness campaigns, the availability of resources, and future projections of the pandemic, people may become more informed and better equipped to handle the psychological effects of the prolonged outbreak. This increased familiarity with the disease and prevention strategies can help individuals manage their response to the situation [20, 21]. A recent survey conducted among healthcare workers revealed that a notable 27.3% believed that political pressure or red tape led them to compromise ethical principles during the pandemic. Furthermore, a significant 34.5% reported that insufficient monetary compensation or work appreciation resulted in compromised care for Covid-19 patients. These results emphasize the importance of addressing these concerns to enable healthcare providers to deliver excellent care without compromising ethical standards.

Further, it has been reported that nurses with higher education perceived less risk of infection, which draws attention to training diploma nurses on different risk prevention strategies, care modules, infection prevention policies, and other safety measures released from time to time in the pandemic. Similar findings are reported in many previously published studies [22, 23].

Although nurses work under highly stressful conditions and are at high risk of contracting infections during the pandemic, they still appreciate their profession and do not regret choosing it as a career. Nurses are highly motivated and provide round-the-clock services to treat patients. These findings are consistent with previous research on the commitment and dedication of nurses to work during emergencies [24, 25]. Further, it is also clear that a high anxiety level will result in poor commitment to organization among nurses [26]. Given the heightened risk posed by the pandemic, nurses must carefully consider the extent of their involvement in patient care while also taking steps to protect themselves [27].

It’s truly reassuring to know that nurses are unwavering in their commitment to uphold ethical principles, even in such a daunting and challenging situation. Patients must be treated impartially and without prejudice, regardless of their age, gender, or medical background. Undoubtedly, nurses are going above and beyond to provide exceptional care while taking measures to ensure their safety during this pandemic [9].

Many people strongly oppose disconnecting life-saving machines, such as ventilators or oxygen supplies, from elderly patients in favor of younger patients. Nurses receive extensive training on ethical principles for patient care across the world [28]. *Utilitarianism* is the principle that the morally right decision is the one that benefits the greatest amount of good for the greatest number of people [29]. *Distributive justice* is another ethical principle that guides the fair distribution of medical resources as needed [9]. *The egalitarian principle* further emphasizes equal treatment of all, regardless of age, and other characteristics [30]. Use of ethical principles by nurses justifies the care to maximize benefits to the critically sick and needy rather than preferring some specific age group patients. The findings reflect a clear understanding to nurses of human lives and their entitlement to healthcare, regardless of life expectancy and outcomes, supported by another finding [3, 31]. Some nurses believe that the “fair innings” principle allows younger patients to have a chance to reach later stages of life over older patients [32]. In this research, most nurses share the approach of providing care to all, regardless of age or other criteria used to limit access to scarce resources during a pandemic. This finding is similar to other studies [31, 33]. Nurses have reported that providing care for Covid-19 patients is an obligation, regardless of the patient’s condition. This finding is similar to another qualitative study [34] that found caring for Covid-19 patients during the pandemic is a professional obligation, providing valuable experience in caring for these patients.

Nurses who worked during the pandemic have high expectations for their organizations to provide them with appropriate support promptly. It’s also unanimous among them that more support should be given during these challenging times. Moreover, a survey conducted on 231 registered nurses in Israel clearly indicated that they didn’t feel adequately protected and supported in their work environment during the pandemic. This makes it imperative for organizations to prioritize the well-being of their healthcare workers, particularly during times of crisis [9]. These findings are supported by another survey conducted on US nurses [35],and 142 nurses from North Iran [36], which reported having no organizational process or resources to comply with their ethical concerns. Healthcare organizations should prioritize nurse well-being during the pandemic to prevent burnout and ensure quality patient care. Providing support and resources can help combat job dissatisfaction and the intention to quit [35, 37]. Studies recommended fostering and enhancing a supportive and ethical work climate to manage better interwoven ethical issues and make rightful decisions [35, 37]. Existing research also highlighted the importance of providing clear and accessible communication about revised clinical protocols, guidelines, and policies as and when required during the pandemic [9, 38]. Healthcare organizations need to make nurse well-being a top priority during the pandemic. By providing support and resources, burnout can be prevented, and the quality of patient care can be maintained. Factors that drive nurses include their knowledge, experience, salary, and autonomy. However, negative attitudes towards the pandemic from society can demotivate nurses, so education and awareness programs can be helpful [39]. Nurses expect that the community should not stigmatize patients and nurses involved in the care of patients with Covid-19 [34].

Nurses, as the largest workforce in the care industry, often experience extensive stress frightening, and struggle a lot during public health emergencies, including Covid-19. Chronic stress can negatively impact both physical and mental health, ultimately affecting the quality of patient care. Healthcare organizations must provide resources and support to prevent burnout and promote mental health. Additionally, education and awareness programs can help combat negative attitudes towards the pandemic, which can demotivate nurses [40]. Covid-19 fear and anxiety can lead to social stigma hindering medical and mental health care access. We must break this stigma for all to receive the care they need [41]. As healthcare professionals, nurses need to take care of their mental health and practice positive coping strategies. Some strategies mentioned in the literature include venting, listening to music, getting adequate rest, meditation, developing a support system with friends, colleagues, and family members, and developing faith in God. These strategies can help support mental health and promote balanced decision-making in future catastrophic situations [42, 43].

## Limitations

It is essential to acknowledge the limitations of this research work, as it was conducted during the pandemic and limited to a single large tertiary care hospital. The response rate to the survey questionnaires could have been more optimal, which may affect the generalization of the findings. Additionally, the survey may cover only some ethical concerns and may need to be explored in future qualitative studies. Finally, the voluntary nature of participation in the study may have introduced selection bias.

## Conclusion

The research findings suggest that nurses face a significant risk of contracting infections during the pandemic. Despite this personal risk, nurses showed great dedication and commitment to their duties. With the possibility of future pandemics, it is essential to raise awareness within communities and improve attitudes towards healthcare workers to boost morale and motivation for longer working hours. Providing appropriate resources, organizational support and promoting healthy coping strategies could be potential solutions to protect the mental health of nurses.

## Data Availability

Data are available with corresponding author and will be available on request of the user.

## References

[1] Chan JFW, Yuan S, Kok KH, To KKW, Chu H, Yang J (2020). A familial cluster of Pneumonia associated with the 2019 novel coronavirus indicating person-to-person transmission: a study of a family cluster. Lancet.

[2] Chamsi-Pasha H, Chamsi-Pasha M, Albar MA (2020). Ethical dilemmas in the era of COVID-19. Avicenna J Med.

[3] Gebreheat G, Teame H (2021). Ethical challenges of nurses in covid-19 pandemic: integrative review. J Multidiscip Healthc.

[4] LNU S, Kumar R (2021). Promoting Mental Health of nurses during the Coronavirus Pandemic: will the Rapid Deployment of nurses’ Training Programs during COVID-19 Improve Self-Efficacy and reduce anxiety?. Cureus.

[5] Lam SKK, Kwong EWY, Hung MSY, Pang SMC, Chien WT (2019). A qualitative descriptive study of the contextual factors influencing the practice of emergency nurses in managing emerging infectious Diseases. Int J Qual Stud Health Well-being.

[6] Emanuel EJ, Persad G, Upshur R, Thome B, Parker M, Glickman A (2020). Fair allocation of Scarce Medical resources in the time of Covid-19. N Engl J Med.

[7] Bansal P, Bingemann TA, Greenhawt M, Mosnaim G, Nanda A, Oppenheimer J (2020). Clinician Wellness during the COVID-19 pandemic: extraordinary Times and Unusual challenges for the Allergist/Immunologist. J Allergy Clin Immunol Pract.

[8] Maben J, Bridges J (2020). Covid-19: supporting nurses’ psychological and mental health. J Clin Nurs.

[9] Sperling D (2021). Ethical dilemmas, perceived risk, and motivation among nurses during the COVID-19 pandemic. Nurs Ethics.

[10] Jourdain G, Chênevert D (2010). Job demands-resources, burnout and intention to leave the nursing profession: a questionnaire survey. Int J Nurs Stud.

[11] Sperling D (2021). Training nurses to Better Deal with ethical dilemmas during pandemics. Disaster Med Public Health Prep.

[12] IBM Corp (2017). IBM SPSS statistics for Windows: Version 23.0.

[13] Nadeem F, Sadiq A, Raziq A, Iqbal Q, Haider S, Saleem F (2021). Depression, anxiety, and stress among nurses during the covid-19 wave iii: results of a cross-sectional assessment. J Multidiscip Healthc.

[14] Arnetz JE, Goetz CM, Arnetz BB, Arble E (2020). Nurse reports of stressful situations during the COVID-19 pandemic: qualitative analysis of survey responses. Int J Environ Res Public Health.

[15] Hu D, Kong Y, Li W, Han Q, Zhang X, Zhu LX (2020). Frontline nurses’ burnout, anxiety, depression, and fear statuses and their associated factors during the COVID-19 outbreak in Wuhan, China: a large-scale cross-sectional study. EClinicalMedicine.

[16] Jose S, Cyriac MC, Dhandapani M, Mehra A, Sharma N (2022). Mental Health outcomes of perceived stress, anxiety, fear and insomnia, and the resilience among Frontline nurses Caring for critical COVID-19 patients in Intensive Care Units. Indian J Crit Care Med.

[17] Karimi Khordeh N, Dehvan F, Dalvand S, Repišti S, Ghanei Gheshlagh R (2022). The COVID-19 fear, anxiety, and resilience among emergency nurses. Front Psychol.

[18] Da Rosa P, Brown R, Pravecek B, Carotta C, Garcia AS, Carson P (2021). Factors associated with nurses emotional distress during the COVID-19 pandemic. Appl Nurs Res.

[19] Kumar R, Das A, Singh V, Gupta P, Bahurupi Y (2021). Rapid survey of psychological status of health-care workers during the early outbreak of COVID-19 pandemic: a single-centre study at a tertiary care hospital in Northern India. J Med Evid.

[20] Dalal PK, Roy D, Choudhary P, Kar SK, Tripathi A (2020). Emerging mental health issues during the COVID-19 pandemic: an Indian perspective. Indian J Psychiatry.

[21] Ghozy S, Cross WM, Islam S, Al-Mawali AH, AlQurashi AA, Hamza A (2022). Psychological impact of COVID-19 on healthcare workers: cross-sectional analyses from 14 countries. Glob Ment Heal.

[22] Kuru Alici N, Ozturk Copur E (2022). Anxiety and fear of COVID-19 among nursing students during the COVID-19 pandemic: a descriptive correlation study. Perspect Psychiatr Care.

[23] LNU S, Kumar R (2021). Promoting Mental Health of nurses during the Coronavirus Pandemic: will the Rapid Deployment of nurses’ Training Programs during COVID-19 Improve Self-Efficacy and reduce anxiety?. Cureus.

[24] Kagan I, Itzhaki M, Melnikov S (2017). Patriotism, organizational commitment and nurses’ intention to report for work in emergencies. Int Nurs Rev.

[25] Melnikov S, Itzhaki M, Kagan I (2014). Israeli nurses’ intention to report for work in an emergency or Disaster. J Nurs Scholarsh.

[26] Son HS, Kim K, Cho IK, Lee J, Choi JM, Kil KH (2022). Healthcare Workers’ Resilience mediates the influence of organizational commitment and anxiety response to viral epidemic on their quality of life in the COVID-19 pandemic. Front Psychiatry.

[27] Kollie ES, Winslow BJ, Pothier P, Gaede D (2017). Deciding to work during the Ebola outbreak: the voices and experiences of nurses and midwives in Liberia. Int J Africa Nurs Sci.

[28] International Council of Nurses. The ICN code of Ethics for nurses: revised 2021. International Council of Nurses; 2021. p. 12. https://www.icn.ch/sites/default/files/2023-06/ICN_Code-of-Ethics_EN_Web.pdf.

[29] Meier LJ (2022). Systemising triage: COVID-19 guidelines and their underlying theories of distributive justice. Med Heal Care Philos.

[30] John TM, Millum J, First Come. First Served? Ethics. 2020;130.

[31] Alloubani A, Khater W, Akhu-Zaheya L, Almomani M, Alashram S (2021). Nurses’ Ethics in the care of patients during the COVID-19 pandemic. Front Med.

[32] Emanuel EJ, Wertheimer A. Who should get Influenza vaccine when not all can? Science (80-). 2006;312:854–5. 10.1126/science.1125347 16690847

[33] Liu X, Xu Y, Chen Y, chen C, Wu Q, Xu H (2022). Ethical dilemmas faced by frontline support nurses fighting COVID-19. Nurs Ethics.

[34] Siregar HR, Simamora FA, Daulay NM, Ritonga SH, Simamora AA, Harahap MA (2022). Nurse’s experience in giving nursing care to Covid-19 patients. Enferm Clin.

[35] Ulrich C, O’Donnell P, Taylor C, Farrar A, Danis M, Grady C (2007). Ethical climate, ethics stress, and the job satisfaction of nurses and social workers in the United States. Soc Sci Med.

[36] Asgari S, Shafipour V, Taraghi Z, Yazdani-Charati J (2019). Relationship between moral distress and ethical climate with job satisfaction in nurses. Nurs Ethics.

[37] Abou Hashish EA (2017). Relationship between ethical work climate and nurses’ perception of organizational support, commitment, job satisfaction and turnover intent. Nurs Ethics.

[38] El Sharif N, Ahmead M, Imam A. COVID-19 Infection prevention and control procedures and institutional trust: perceptions of Palestinian healthcare workers. Front Public Health. 2022. 10. 10.3389/fpubh.2022.947593 PMC9437519 36062099

[39] Nashwan AJ, Valdez GFD, AL-Fayyadh S, Al-Najjar H, Elamir H, Barakat M (2022). Stigma towards health care providers taking care of COVID-19 patients: a multi-country study. Heliyon.

[40] Chan AOM, Chan YH (2004). Psychological impact of the 2003 severe acute respiratory syndrome outbreak on health care workers in a medium size regional general hospital in Singapore. Occup Med (Chic Ill).

[41] Xiang YT, Yang Y, Li W, Zhang L, Zhang Q, Cheung T (2020). Timely mental health care for the 2019 novel coronavirus outbreak is urgently needed. The Lancet Psychiatry.

[42] Munawar K, Choudhry FR (2021). Exploring stress coping strategies of frontline emergency health workers dealing Covid-19 in Pakistan: a qualitative inquiry. Am J Infect Control.

[43] Cui S, Jiang Y, Shi Q, Zhang L, Kong D, Qian M (2021). Impact of covid-19 on anxiety, stress, and coping styles in nurses in emergency departments and Fever clinics: a cross-sectional survey. Risk Manag Healthc Policy.

